# Three-years misdiagnosis of Niemann Pick disease type B with novel mutations in *SMPD1* gene as Budd-Chiari syndrome

**DOI:** 10.1186/s12920-022-01353-2

**Published:** 2022-09-16

**Authors:** Zhe-wen Zhou, Shou-hao Wang, Cheng-an Xu, Wen-hao Wu, Tian-chen Hui, Qiao-qiao Yin, Wei Zheng, Hong-ying Pan

**Affiliations:** 1 Graduate School of Clinical Medicine, Bengbu Medical College, Bengbu, 233000 Anhui Province China; 2 Department of Infectious Diseases, Center for General Practice Medicine, Zhejiang Provincial People’s Hospital (Affiliated People’s Hospital, Hangzhou Medical College), Hangzhou, 310014 Zhejiang Province China

**Keywords:** Niemann Pick disease type B, Sea-blue histiocytosis, Budd-Chiari syndrome, *SMPD1* gene, Case report

## Abstract

**Background:**

The chronic visceral subtype of acid sphingomyelinase deficiency, commonly known as Niemann Pick disease type B (NPDB), is a relatively rare autosomal recessive genetic disorder that is caused by mutations in the *SMPD1* gene. NPDB with sea-blue histiocytes (SBH) clinically mimics Budd-Chiari syndrome (BCS), as it lacks specific clinical characteristics. This makes its diagnosis difficult.

**Case presentation:**

Here, we report a case of NPDB with SBH that was misdiagnosed as BCS for three years. A 20-year-old female with abdominal distension, hepatosplenomegaly, and haematological anomalies was initially diagnosed with BCS based on her imaging finding of a thin hepatic vein and rapid blood flow at the confluence of the hepatic vein and inferior vena cava. Her bone marrow cytology found sea-blue histiocytes. Liver biopsy showed foamy cytoplasm in hepatocytes surrounded by numerous Kupffer cells. Sequencing analysis of the *SMPD1* gene led to the finding of two missense mutations in the heterozygous state: C.829 T > C (p.Trp277Arg) in exon 2 (novel) and c.1805G > A (p.Arg602His) in exon 6 (already described). These findings established the diagnosis of NPDB.

**Conclusion:**

The patient presented with hepatosplenomegaly, haematological anomalies, and dyslipidaemia. Thus, NPDB should be considered following the exclusion of related diseases. The diagnosis of NPDB was suspected by clinical symptoms and routine laboratory tests and was confirmed by liver biopsy and gene sequencing. The novel mutation c.829 T > C in exon 2 of the *SMPD1* gene has never been reported and needs to be further investigated.

## Background

The chronic visceral subtype of acid sphingomyelinase deficiency, also called Niemann Pick disease type B (NPDB), is a rare autosomal recessive hereditary disease that is caused by mutations in the *SMPD1* gene. *SMPD1* encodes acid sphingomyelinase. Sphingomyelin is abnormally deposited in the liver, spleen, and other parts of the body, and this results in the formation of Niemann-Pick cells that cause hepatosplenomegaly, dyslipidaemia, and haematological anomalies [1]. The most common initial occurrence of NPDB is hepatosplenomegaly, which is usually found in early childhood. Hepatosplenomegaly may be severe, as it can cause the spleen volume to be ten times larger than normal [2]. In addition to hepatosplenomegaly, patients often have abnormal haematology, elevated levels of serum triglycerides and low*-*density lipoprotein cholesterol, interstitial lung disease, and a decreased lung diffusion capacity [3]. Decreased high-density lipoprotein cholesterol levels are due to the abnormal cells that are present in the liver and spleen. Thrombocytopenia and leukopenia usually worsen over time. The diagnosis of NPDB is biochemically based on the level of acid sphingomyelinase activity, which is less than 15% of normal [4]. In addition, genetic sequencing can also aid in the diagnosis of NPDB.

In contrast, Budd-Chiari Syndrome (BCS) refers to a group of heterogeneous diseases in which portal hypertension caused by obstruction of the hepatic venous outflow at the level of hepatic veins or the inferior vena cava, and BCS is associated with the clinical characteristics of abdominal distension, ascites, hepatosplenomegaly, and lower limb oedema. The diagnosis of BCS is established with unequivocal radiological confirmation of hepatic venous outflow obstruction. Doppler ultrasound has a diagnostic sensitivity of over 75% [5]. Both NPDB and BCS have a common clinical characteristic of hepatosplenomegaly, but ultrasound with Doppler may help differentiate between these two conditions. However, it cannot differentiate them from other diseases with hepatic venous outflow tract obstruction.

Sea-blue histiocytosis (SBH) is also a morphological discovery that is related to hereditary or acquired lipid metabolism disorders. It is primarily caused by the congenital deficiency of sphingomyelinase and is secondarily related to abnormal enzyme levels, excessive lipid metabolism, or increased cellular metabolism. Many diseases, including NPDB, primary thrombocytopenic purpura, chronic myeloid leukaemia, myelodysplastic syndrome, thalassemia, and lipoproteinemia, can also be secondary to SBH. The accumulation of sphingomyelin and other lipids forms sea-blue histiocytes. When large number of sea-blue histiocytes infiltrate organs, they can cause splenomegaly, hepatomegaly, anaemia, thrombocytopenic purpura, and other conditions. Sea-blue histiocytes found in the bone marrow smear may further indicate NPDB.

A case of NPDB with novel mutations in the *SMPD1* gene was misdiagnosed as BCS for three years and is reported here.

## Case presentation

A 20-year-old female came to our hospital because of repeated, gradually progressing severe abdominal distension over ten years. No obvious abdominal pain, diarrhoea, or fever occurred. The patient frequently visited the local hospital and was diagnosed with BCS with no abdominal distension relief. She denied any family history of genetic disease, and the marriage of her parents was not consanguineous. She had normal intelligence and demonstrated moderate academic performance.

**Fig. 1:**
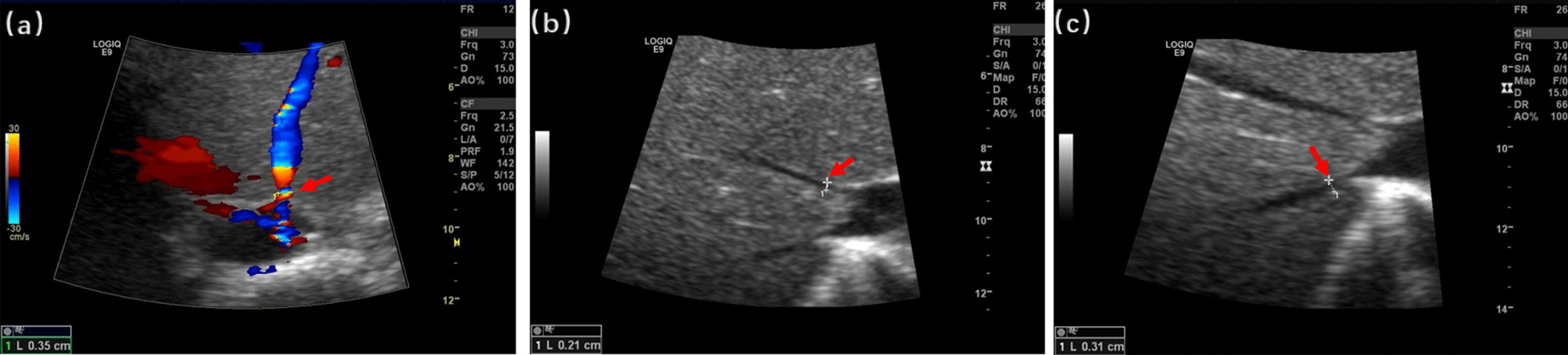
Ultrasound showed that the inner diameter on the left, middle, and right hepatic vein joining the inferior vena cava was 3.5 mm (**a**), 2.1 mm (**b**), and 3.1 mm (**c**)

**Fig. 2:**
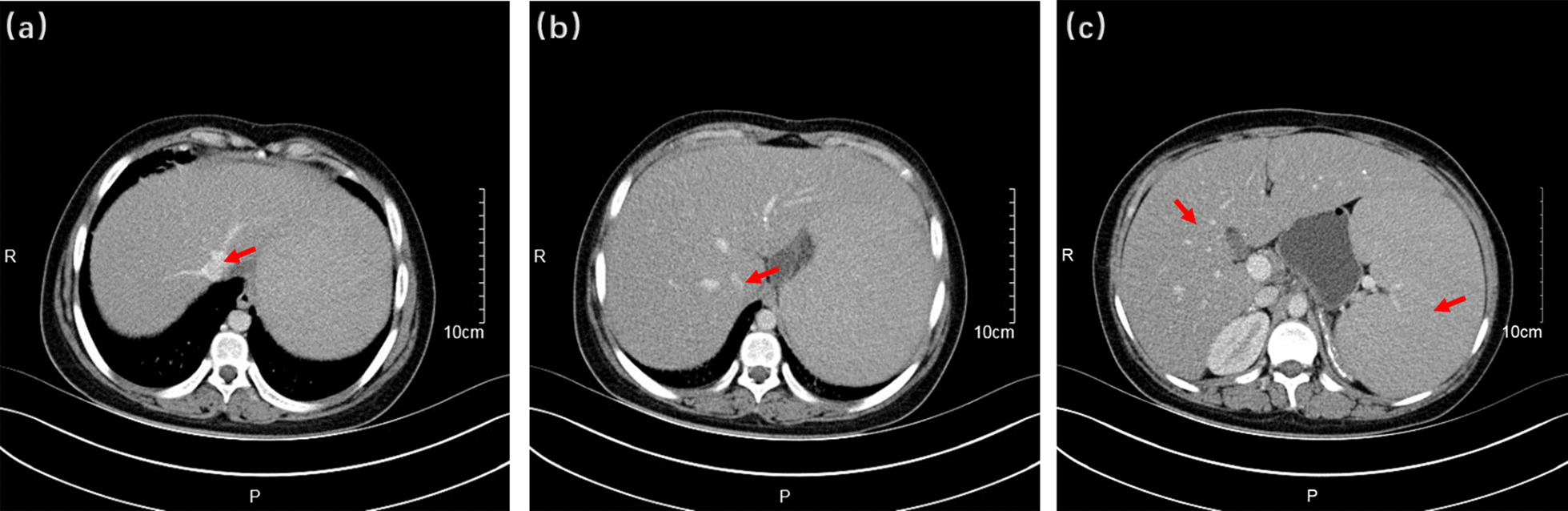
Enhanced CT of the upper abdomen revealed the place where the hepatic vein merged into the inferior vena cava was thin (**a**, **b**) and hepatosplenomegaly (**c**).

**Fig. 3:**
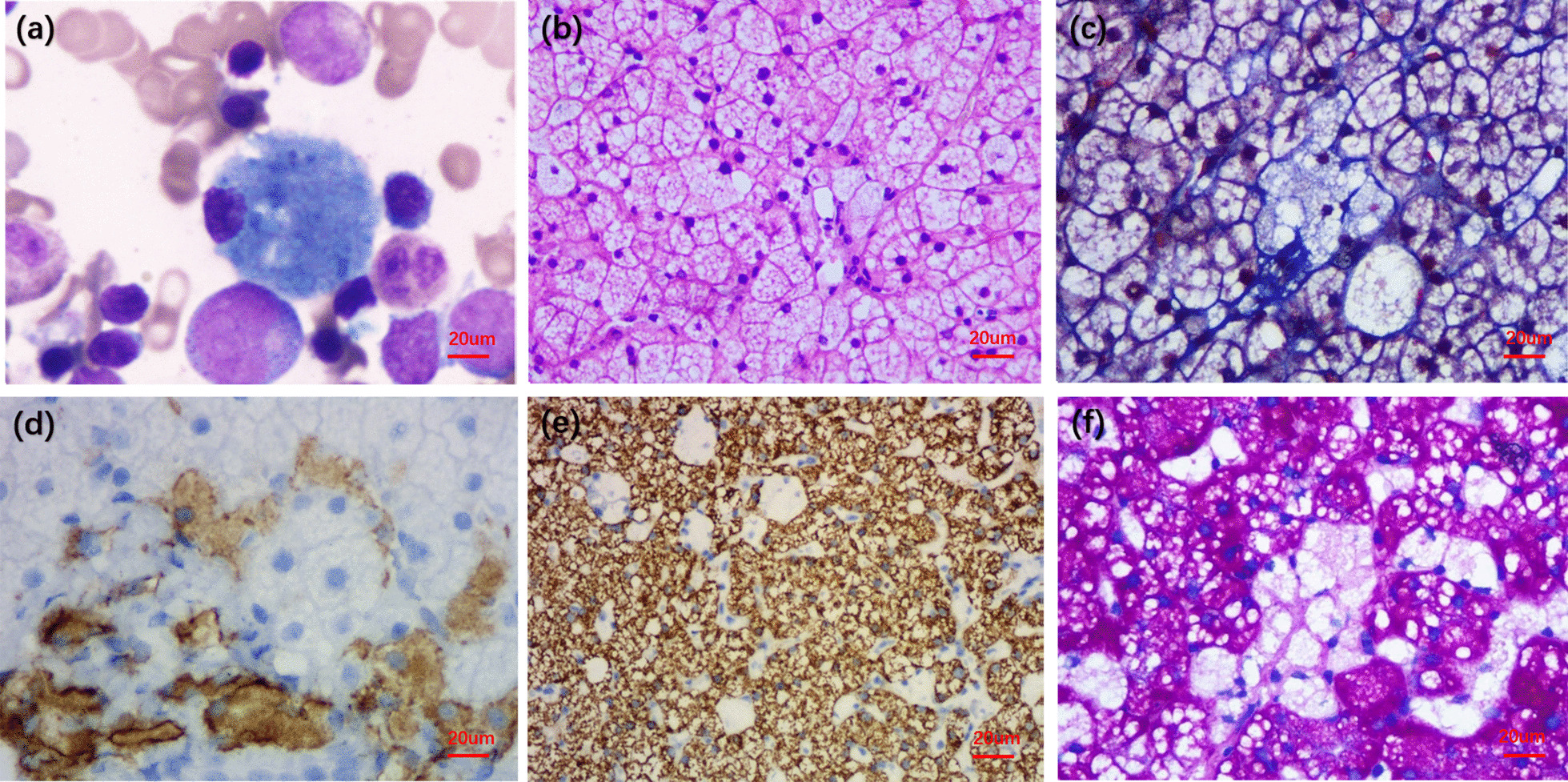
**a**. Smears of bone marrow aspiration revealed sea-blue histiocytes. (Wright–Giemsa’s stain, × 400) **b**. The liver puncture smear showed cytoplasm was foamy. (hematoxylin–eosin staining, × 400) **c**. Masson stain showed hyperplastic fibrous tissue (blue)in liver lobules (× 400) **d**. Immunohistochemical stain for CD68 on Kupffer cells (× 400) **e**. Immunohistochemical stain for Hepatocyte(× 200): Hepatocytes(+); Kupffer cells (−). **f**. Periodic Acid-Schiff stain(× 400): Hepatocytes (+); Kupffer cells (−). (The equipment is biological microscope XSP-11CA)

**Fig. 4:**
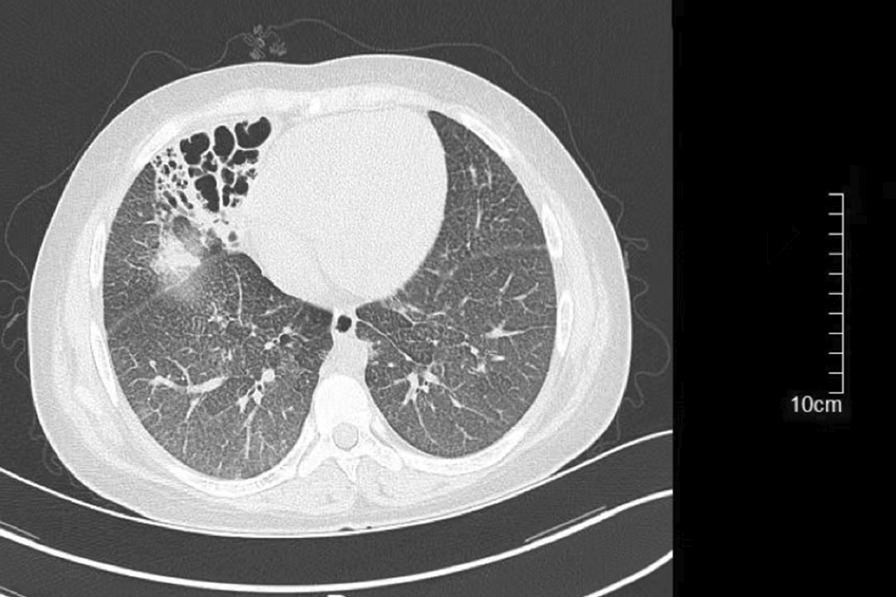
In the lower lobe of the right lung, scattered patchy and cordlike shadows with increased density were observed, and some edges were blurred in CT. The interlobular septum of the lung was thickened in a grid shape, which was consistent with interstitial manifestations

**Fig. 5:**
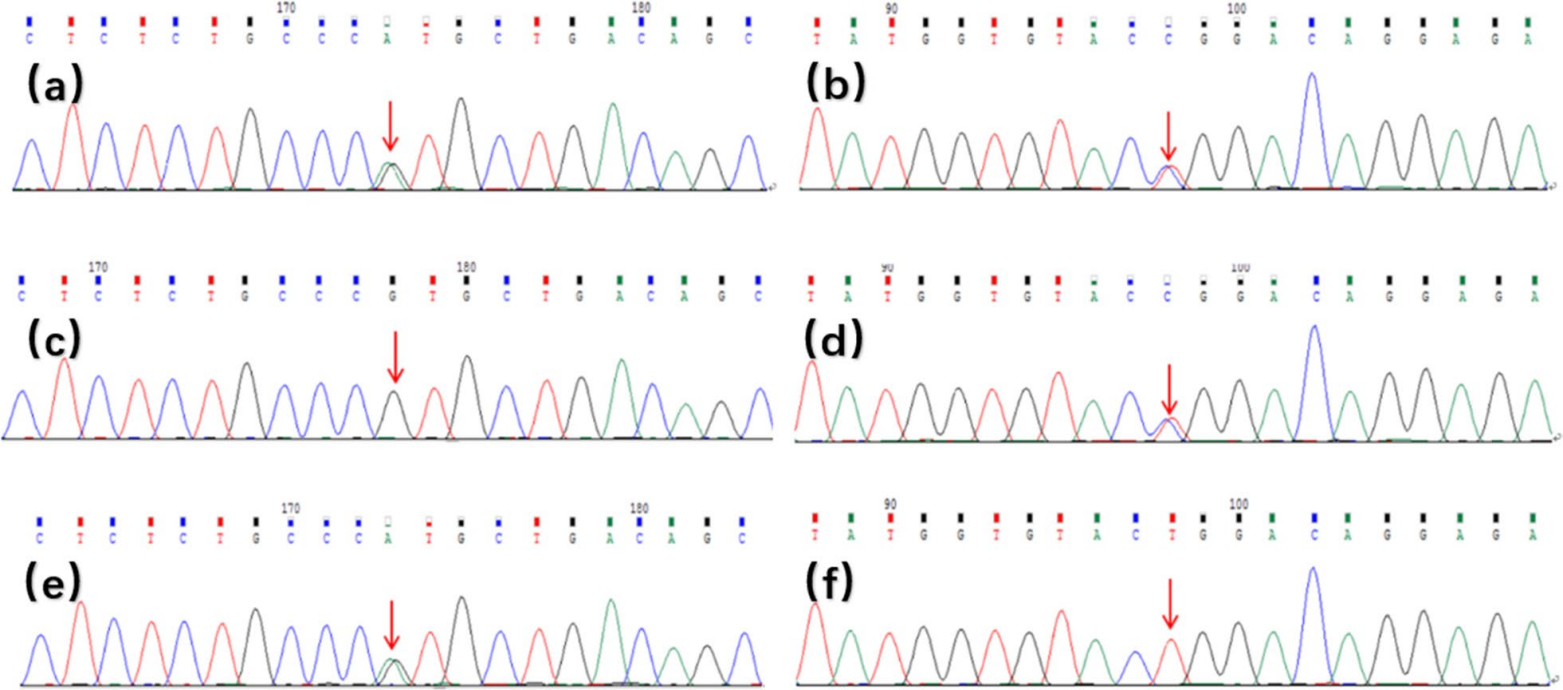
**a**. The patient’s *SMPD1*_ex6 c. c.1805G > A(p. R602H)Sanger. **b**. The patient’s S-MPD1_ex2 c.829 T > C(p. W277R)Sanger. **c**. The patient’s father *SMPD1*_ex6 c. c.1805G > A(p. R602H)Sanger. **d**. The patient’s father *SMPD1*_ex2 c.829 T > C(p. W277R)Sanger. **e**. The patient’s mother *SMPD1*_ex6 c. c.1805G > A(p. R602H)Sanger. **f**. The patient’s mother *SMPD1*_ex2 c.829 T > C(p. W277R)Sanger. **a**,** c**,** e**. NM_000543.4 sequence: CTCTCTGCCCGTGCTGACAGC. **b**,** d**,** f**. NM_000543.4 sequence: TATGGTGTACTGGACAGGAGA (This mutation naming rule refers to Human Genome Variation Society.)

Physical examination showed that the liver was enlarged 15 cm below the ribs, with a medium texture and no tenderness. The spleen was 20 cm, 20 cm, and -1 cm in size at the lines of I, II, and III, respectively, with medium texture and no tenderness. There were no exposed abdominal veins and no oedema in the lower limbs. Clinical laboratory testing results were as follows: leukocytes, 2.17 × 10^^^9/L; red blood cells, 3.53 × 10^^^12/L; platelets, 80 × 10^^^9/L; haemoglobin, 73 g/L; mean red blood cell volume, 72 fl; mean cell haemoglobin, 20.7 pg; aspartate-aminotransferase, 44 U/L; alanine-aminotransferase, 37 U/L; gamma-glutamyl transpeptidase, 19 U/L; alkaline phosphatase, 119 U/L; total bilirubin, 43.0 µmol/L; indirect bilirubin, 36.5 µmol/L; triglycerides, 2.53 mmol/L; high-density lipoprotein cholesterol, 0.35 mmol/L; and low-density lipoprotein cholesterol, 3.74 mmol/L. Abdominal ultrasonography with venous Doppler revealed rapid blood flow at the confluence of the hepatic vein and inferior vena cava. The inner diameters of the left, middle, and right hepatic veins joining the inferior vena cava were 3.5 mm, 2.1 mm, and 3.1 mm, respectively (Fig. 1). The upper abdominal enhanced CT scan showed hepatosplenomegaly, and thinner hepatic veins merged into the inferior vena cava (Fig. 2). Bone marrow examination confirmed the presence of sea-blue histiocytes (Fig. 3). Based on the morphological findings, liver biopsy and gene sequencing were performed. Immunohistochemical staining revealed foamy cytoplasm in the hepatocytes surrounded by a large number of Kupffer cells. Periodic acid-Schiff staining further confirmed this finding (Fig. 3). Pulmonary CT showed that the pulmonary interlobular septum was thickened in a grid shape, which was consistent with interstitial manifestations (Fig. 4). Sanger sequencing of the *SMPD1* gene revealed two heterozygous missense mutations, c.1805G > A (p.Arg602His) in exon 6 and c.829 T > C (p.Trp277Arg) in exon 2 (Fig. 5). A parental study demonstrated allele segregation. p.Arg602His was of maternal origin, and p.Trp277Arg was of paternal origin (Fig. 5).

Symptomatic therapies with liver function protection, diuresis, and iron supplementation were followed after admission. Warfarin was used for anticoagulant therapy of BCS, and the dose was adjusted according to the INR value. The patient was discharged after the abdominal distension and other symptoms were relieved. We have followed up with this patient thus far. The patient is now 24 years old.

## Discussion and conclusion

Classically, the diagnosis of NPDB biochemically relies on the determination of acid sphingomyelinase activity in leukocytes, dried blood spots or cultured skin fibroblasts. Activity in patients is less than 15% of the normal value [4]. However, we were unable to measure acid sphingomyelinase activity in our patient because too few laboratories currently offer this assay. Therefore, advanced determination methods, such as bone marrow cell biology and gene sequencing analysis, are helpful in further clarifying the final diagnosis. In this case, we report that the patient was misdiagnosed with BCS for three years because the diagnosis was based only on the clinical characteristics of hepatosplenomegaly and hepatic vein imaging. Gene sequencing analysis with parental study further determined the final diagnosis of NPDB. The c.1805G > A (p.Arg602His) mutation in exon 6 of the *SMPD1* gene has been reported in the relevant scientific literature [6, 7]. It has been included in the ClinVar database. In a study of 118 Chinese patients with NPA/B, 92 different SMPD1 variants were identified. The most prevalent mutation was p.Arg602His, which accounted for 9.3% of alleles [8]. We also found another mutation locus, c.829 T > C, in exon 2 of the *SMPD1* gene. The pathogenicity of this locus has not yet been reported, and the frequency of this mutation locus in the normal population is zero. SIFT and Polyphen-2 predicted the novel mutation to be disease causing.

Hu et al. found a link between BCS and SBH in one case, but the patient refused liver biopsy. Thus, whether SBH was the exact cause of BCS could not be determined. SBH can be a secondary cause of NPDB, BCS, or multiple myeloma, which may make this case more complicated in clinical settings [9]. There is also a link between BCS and lipid storage diseases. Some researchers have found that lipid storage diseases should be included as a risk factor for BCS. They found that three Egyptian children with BCS had lipid storage diseases, of which one is Niemann Pick Disease [10]. Gaucher’s cells focally infiltrate the liver parenchyma at the hepatic venous confluence. This compromises the hepatic veins and the inferior vena cava and causes BCS [11]. The above connections provide us with ideas for differential diagnosis in many aspects. In this case, the major clinical characteristics are abdominal distension and hepatosplenomegaly, which make it difficult to differentiate NPDB from BCS without further advanced examinations. Thus, it had been misdiagnosed as BCS for three years.

Imaging findings showed that the thinner hepatic vein merged into the inferior vena cava with increased blood flow following her admission. Hepatosplenomegaly can be observed in a variety of diseases, including NPDB, BCS, Gaucher disease, leukaemia, lymphoma, multiple myeloma, hepatic venous-occlusive disease, and primary liver tumours. If patients present with hepatosplenomegaly, haematological anomalies, and dyslipidaemia, the diagnosis should be cautiously considered. Further advance examinations, such as bone marrow cell biology and gene sequencing analysis, should be performed to reduce misdiagnoses and to provide an optimal therapeutic option. The novel mutation site (c.829 T > C in exon 2 of the *SMPD1* gene) in this case has not been reported in the literature thus far and needs to be further confirmed. Of note, specific enzyme replacement therapy for NPDB may soon become available since clinical trials using olipudase alfa in adult and paediatric patients have shown significant improvements across several clinically relevant endpoints, particularly spleen size and DLCO [12–14]. This treatment, however, is currently not approved by regulatory agencies, except in Japan. In the future, specific enzyme replacement therapy with NPDB deserves our attention.

## Data Availability

The data analysed in this study is deposited in NCBI GenBank. The accession number is OP185930.

## Abbreviations

NPDB: Niemann Pick disease type B
SBH: Sea-blue histiocytes
BCS: Budd-Chiari syndrome
SIFT: Sorts intolerant from tolerant
PolyPhen-2: Polymorphism phenotyping v2
DLCO: Diffusion lung capacity for carbon monoxide
